# Elevated extracellular calcium ions promote proliferation and migration of mesenchymal stem cells via increasing osteopontin expression

**DOI:** 10.1038/s12276-018-0170-6

**Published:** 2018-11-05

**Authors:** Mi Nam Lee, Hee-Su Hwang, Sin-Hye Oh, Amir Roshanzadeh, Jung-Woo Kim, Ju Han Song, Eung-Sam Kim, Jeong-Tae Koh

**Affiliations:** 1 Research Center for Biomineralization Disorders, School of Dentistry, Chonnam National University, Gwangju, Republic of Korea; 2 Department of Pharmacology and Dental Therapeutics, School of Dentistry, Chonnam National University, Gwangju, Republic of Korea; 3 School of Biological Sciences and Biotechnology, Chonnam National University, Gwangju, Republic of Korea; 4 Department of Biological Sciences, Chonnam National University, Gwangju, Republic of Korea

**Keywords:** Mesenchymal stem cells, Cell signalling

## Abstract

Supplementation of mesenchymal stem cells (MSCs) at sites of bone resorption is required for bone homeostasis because of the non-proliferation and short lifespan properties of the osteoblasts. Calcium ions (Ca^2+^) are released from the bone surfaces during osteoclast-mediated bone resorption. However, how elevated extracellular Ca^2+^ concentrations would alter MSCs behavior in the proximal sites of bone resorption is largely unknown. In this study, we investigated the effect of extracellular Ca^2+^ on MSCs phenotype depending on Ca^2+^ concentrations. We found that the elevated extracellular Ca^2+^ promoted cell proliferation and matrix mineralization of MSCs. In addition, MSCs induced the expression and secretion of osteopontin (OPN), which enhanced MSCs migration under the elevated extracellular Ca^2+^ conditions. We developed in vitro osteoclast-mediated bone resorption conditions using mouse calvaria bone slices and demonstrated Ca^2+^ is released from bone resorption surfaces. We also showed that the MSCs phenotype, including cell proliferation and migration, changed when the cells were treated with a bone resorption-conditioned medium. These findings suggest that the dynamic changes in Ca^2+^ concentrations in the microenvironments of bone remodeling surfaces modulate MSCs phenotype and thereby contribute to bone regeneration.

## Bone formation: released calcium could stimulate stem cells

Studies with mouse cells and bone samples indicate how calcium ions released during the normal turnover of bone promote the supplementation of stem cells needed to make new bone during routine “remodeling” and regeneration after injury. Our bones are degraded and remodeled continuously, requiring the controlled supplement of stem cells that create bone-forming cells called osteoblasts. Jeong-Tae Koh and colleagues at Chonnam National University, Gwangju, South Korea, exposed the relevant stem cells to increased concentrations of external calcium ions. This promoted cell proliferation and migration that allow the cells to support new bone formation. The research suggests this effect on stem cells may be achieved by calcium ions released directly from bone as it is naturally degraded. The findings may help researchers develop new ways to encourage bone regeneration after injury and surgery.

## Introduction

Bone is remodeled throughout adult life not only to regulate mineral homeostasis but also to maintain the integrity and biomechanical stability of the skeleton^1^. Bone remodeling is accomplished through tightly regulated and continuous cycles of osteoclastic and osteoblastic activity in the bone matrix^2^. This process requires osteoblast mobilization to the sites of bone reconstruction. However, because osteoblasts are non-proliferative and have a short lifespan, the replenishment of osteoblasts from MSCs is required for continuous bone formation^3^. Indeed, defects in MSCs recruitment are associated with several skeletal pathologies including osteoporosis^4,5^. Therefore, stimulation of MSCs recruitment to sites of bone formation represents a promising strategy for bone regeneration.

During osteoclast-mediated bone resorption, multiple factors released from bone matrix locally are known to create an osteogenic microenvironment that promotes MSCs recruitment and osteoblast differentiation resulting in new bone formation. The osteoclastic bone resorptive sites contain several soluble factors, including transforming growth factor β1 (TGFβ1) and insulin growth factor 1, which act as a chemoattractant to induce cell migration in vitro^6–8^. TGFβ1 is one of the most abundant cytokines in the bone matrix, which is released and activated during osteoclast-mediated bone resorption^6,9–11^. Previously, using an in vivo mouse tibial fracture model, TGFβ1 was suggested to be critical in the recruitment of MSCs to bone remodeling sites by mediating Smad signaling pathway^12^. Other growth factors and cytokines are also reported to regulate MSCs migration in vitro so far^13^.

Calcium ions (Ca^2+^) are released from bone matrix during osteoclast-mediated bone resorption, although the exact concentration of extracellular Ca^2+^ during bone remodeling in vivo is still unknown. The resorptive action of osteoclasts results in a local increase of extracellular Ca^2+^ concentration as high as 40 mM in vitro^14^. Another study showed that the extracellular Ca^2+^ concentrations between the basal aspect of cells and substrate of damage zones of bone can increase to 10 mM within sec, suggesting fluctuation within the range of 9–180 mM at the sites of damage in vivo^15^. Indeed, the effect of elevated extracellular Ca^2+^ on osteoblasts-mediated bone formation has been evaluated^16–19^. Many studies have focused on the effect of extracellular Ca^2+^ on committed osteoblasts, while a few recent reports showed that Ca^2+^ concentration also influenced MSCs phenotypes, including cell proliferation and differentiation^20–24^. However, the direct effect of Ca^2+^ released from bone resorption surfaces on MSCs function has not been evaluated, and moreover, the role of extracellular Ca^2+^ in MSCs migration is largely unknown.

In this study, we investigated the role of elevated extracellular Ca^2+^ in MSCs phenotype alterations and the effects of Ca^2+^ released from bone resorption surface on MSCs behavior. We suggest that the elevated extracellular Ca^2+^ represents a critical factor in the expansion of MSCs population in bone remodeling sites by activating cell proliferation and migration.

## Materials and methods

### Reagents

We used the recombinant mouse OPN, TGFβ1, and FGF2 supplied by R&D Systems (Minneapolis, MN, USA). Recombinant human bone morphogenetic protein 2 (BMP2) was purchased from CowellMedi (Busan, South Korea). Quantikine Mouse/Rat osteopontin, Mouse/Rat/Porcine/Canine TGFβ1, and Mouse/Rat FGF basic ELISA kits were obtained from R&D Systems. Cell Counting Kit-8 (CCK-8) assay kit was supplied by Dojindo Laboratories (Kumamoto, Japan), and BrdU incorporation assay kit was ordered through Cell Signaling Technology (Danvers, MA, USA). The molecular biology-grade reagents were purchased from Sigma-Aldrich (St. Louis, MO, USA) unless stated otherwise.

### Cell culture

C3H10T1/2 cells obtained from the American Type Culture Collection (ATCC, Manassas, VA, USA) were maintained in Dulbecco’s modified Eagle’s medium (DMEM) (Gibco/Thermo Fisher Scientific, Waltham, MA, USA) containing 10% fetal bovine serum (FBS) (Gibco/Thermo Fisher Scientific), supplemented with 100 U/mL of penicillin and 100 µg/mL of streptomycin (Invitrogen/Thermo Fisher Scientific). Mouse bone marrow MSCs (BM-MSCs) were isolated from the femurs of 6–8-week-old male C57BL/6 mice as previously described^25^. BM-MSCs were cultured in α-minimal essential medium (α-MEM) (Gibco/Thermo Fisher Scientific) supplemented with 10% FBS, 100 U/mL of penicillin, and 100 µg/mL of streptomycin. We used BM-MSCs at passage 3 for further experimentation.

### Cell proliferation assay

Cells were incubated with 2% FBS/medium containing the indicated amount of CaCl_2_ for 48 h. The following media containing increasing extracellular Ca^2+^ concentrations were used for this study: 1.8 mM Ca^2+^ (standard culture medium, SM, contains 1.8 mM CaCl_2_), 4 mM Ca^2+^ (SM + 2.2 mM CaCl_2_), 6 mM Ca^2+^ (SM + 4.2 mM CaCl_2_), 8 mM Ca^2+^ (SM + 6.2 mM CaCl_2_), 10 mM Ca^2+^ (SM + 8.2 mM CaCl_2_), and 20 mM Ca^2+^ (SM + 18.2 mM CaCl_2_). For OPN neutralizing antibody assays, the indicated concentration of OPN neutralizing antibody (R&D Systems) was added to elevated extracellular Ca^2+^ medium. Cell proliferation was determined using a CCK-8 assay or a BrdU incorporation assay as previously described^26^. Relative proliferation rates were determined by normalizing the absorbance value of the experimental groups to that of control group. Values for the relative proliferation rates represented as the mean ± SEM of triplicate reactions in one representative experiment.

### Real-time PCR

Total RNA was isolated from cells using TRIzol reagent (Invitrogen/Thermo Fisher Scientific) and 1 µg of total RNA was reverse transcribed using a reverse transcription system (200 U of M-MLV, 0.5 mM of dNTP, 40 U of RNAsin, and 200 ng of random primer) (Promega, Madison, WI, USA), according to the manufacturer’s protocol. We used the StepOnePlus Real-Time PCR System (ABI, Abilene, TX, USA) for quantitative real-time PCR. PCR was carried out in a final volume of 20 µL using 10 pmol of each primer (listed below), 5 µL of cDNA (5 ng/µL), and 10 µL of Power SYBR Green PCR Master Mix (ABI, Valencia, CA, USA). PCR was performed with a 5 min pre-incubation period at 95 °C, followed by 40 cycles of 30 s each at 95 °C, 30 s at 56 °C, and 30 s at 72 °C. We performed all reactions in triplicate, and normalized the expression levels of all mRNAs to the expression level of endogenous β-actin. The relative target gene expression was quantified using the comparative Ct method. Values for the relative expression of the indicated genes represent the mean ± SEM of triplicate reactions in one representative experiment. The sequences of the primers used were as follows: for β-actin, 5′-GCCATCTCCTGCTCGAAGTC-3′ and 5′-ACCCACACTGTGCCCATCTA-3′; for FGF2, 5′-CACCAGGCCACTTCAAGGA-3′ and 5′-GATGGATGCGCAGGAAGAA-3′; for TGFβ1, 5′-AACAATTCCTGGCGTTACCTT-3′ and 5′-ATTCCGTCTCCTTGGTTCAG-3′; for OPN, 5′-GATTTGCTTTTGCCTGTTTGG-3′ and 5′-TGAGCTGCCAGAATCAGTCACT-3′

### Western blotting analysis

Total protein was extracted from the cells with lysis buffer (Cell Signaling Technology) and the protein concentration was measured using the BCA protein assay reagent (Bio-Rad Laboratories, Hercules, CA, USA). A protein concentration of 20–30 µg was used to analyze the protein expression level using primary antibodies against OPN (Santa Cruz Biotechnology, Dallas, TX, USA), cyclin D1 (Cell Signaling Technology), c-Jun (Cell Signaling Technology), and β-actin (Santa Cruz Biotechnology, Dallas, TX, USA). After incubation with secondary horseradish peroxidase–conjugated anti-mouse or anti-rabbit antibodies (Thermo Fisher Scientific), we visualized the signals using an enhanced chemiluminescence reagent (ECL; Millipore, Billerica, MA, USA) in a LAS-4000 lumino-image analyzer system (Fujifilm, Tokyo, Japan), followed by semiquantitative evaluation of the bands using densitometry (Multi Gauge V3.0, Fujifilm). The blotting results are representative of three independent experiments.

### Enzyme-linked immunosorbent assay (ELISA)

Quantitative analysis of OPN, TGFβ1, and FGF2 in conditioned medium was performed by ELISA according to the manufacturer’s instructions. Active TGFβ1 was not detected in any conditioned medium before the activation of the latent TGFβ1 by sequential treatment with 1 N HCl and 1.2 N NaOH/0.5 M HEPES. Absorbance was measured by a plate reader (Thermo Fisher Scientific). Values for the proteins levels were expressed as the mean ± SEM of a duplicate reaction of one representative experiment.

### Osteoblast differentiation

Cells were seeded on 48-well culture plates at 5 × 10^4^ cells/well. After 24 h, cells were changed with OM (50 µg/mL of ascorbic acid, 5 mM of β-glycerophosphate, and 100 ng/mL of BMP2) containing the indicated amount of extracellular Ca^2+^. The medium was replaced every 3 days for 16 days. To evaluate matrix mineralization, cells were fixed with 70% cold ethanol and treated with 40 mM of alizarin red solution (pH 4.2) for 30 min. The stained culture plates were scanned with an Epson Perfection V700 (Epson Korea, Seoul, South Korea), and the stained cells were observed via optical microscopy (magnification: 50×, Leica Microsystems, Wetzlar, Germany). The alizarin red stain was extracted with 10% (w/v) cetylpyridinium chloride in 10 mM of sodium phosphate (pH 7.0) for 15 min and absorbance were measured by a spectrophotometry (Thermo Fisher Scientific).

### Cell migration assay

Cell migration assay was evaluated using a 24-well Transwell chamber (8 μm pores, SPL, South Korea). Briefly, 2% FBS culture media containing the indicated amount of extracellular Ca^2+^ were loaded in the lower chambers. C3H10T1/2 or BM-MSCs (2 × 10^4^ cells/well) were suspended in serum-free standard medium, and added to the upper chamber. To neutralize secreted OPN, the indicated culture medium was pre-incubated with an OPN neutralizing antibody (R&D Systems) for 1 h and used as the lower chamber medium. After incubation for the indicated durations, cells migrating to the opposite side of the membrane were fixed with 4% paraformaldehyde and stained with 0.05% crystal violet in 2% ethanol. The migrated cells were counted at least in five fields using a light microscope. Data represent the number of total migrated cells per field of view (FV, x50 magnification) or as fold increase related to that of control.

### Preparation of bone resorption-conditioned medium (BRCM)

To prepare the osteoclastic BRCM, osteoclast differentiation was induced from bone marrow-derived macrophages (BMMs) on the surface of mouse calvarial bone slices. To prepare bone slices, 6–8-week-old male C57BL/6 mice were sacrificed, and a 5-mm-diameter calvarial bone was cut off from each side of the calvariae using a trephine bur (Fine Science Tools, Foster City, CA). Any adherent tissue and periosteum were cleaned off with a scalpel. Bone slices were washed with PBS containing 0.1% Triton X-100 several times. After washing with PBS, bone slices were stored in 70% ethanol under frozen condition until use. BMMs were isolated from femurs of 6-week-old C57BL/6 mice and cultured in α-MEM containing 10% FBS and 30 ng/mL of recombinant M-CSF (R&D Systems) for 3 days. Cells were collected and 5 × 10^4^ cells were plated on the prepared calvarial bone slice. To induce osteoclast differentiation, cells were cultured in the presence of RANKL (100 ng/ml, R&D Systems) and M-CSF (30 ng/ml). The precursor cell-conditioned medium (PCM) was prepared by culturing the cells in the presence of M-CSF (30 ng/ml). The culture medium without BMMs was used as a negative control (CCM). The conditioned medium was collected and was replaced with fresh medium every other day for 16 days. We selected 4-day interval samples from each group and used these to measure extracellular Ca^2+^ concentrations or to perform further experiments. The cultured cells were stained for tartrate-resistant acid phosphatase (TRAP) activity (TRAP staining kit, Sigma-Aldrich). To confirm the topography of excavations made by osteoclasts in calvarial bone slices, the bone surface was analyzed using scanning electron microscopy (SEM) (Hitachi SU-70, Tokyo, Japan).

### Measurement of extracellular calcium concentration

The quantity of calcium ions released from the calvarial bone slices was determined using a calcium colorimetric assay kit (BioVision, Milpitas, CA, USA). The conditioned medium prepared as described above was analyzed according to the manufacturer’s instructions. Values represent the mean ± SEM of a triplicate reaction of one representative experiment.

### Statistical analyses

All experiments were repeated at least three times independently. Data were analyzed with Prism5 (GraphPad, La Jolla, CA, USA) for statistical significance and expressed as mean ± SEM. Statistical analysis was performed by one-way ANOVA followed by Tukey post-hoc test unless stated otherwise.

## Results

### Extracellular Ca^2+^ is an important factor for determining MSCs phenotype

**Fig. 1:**
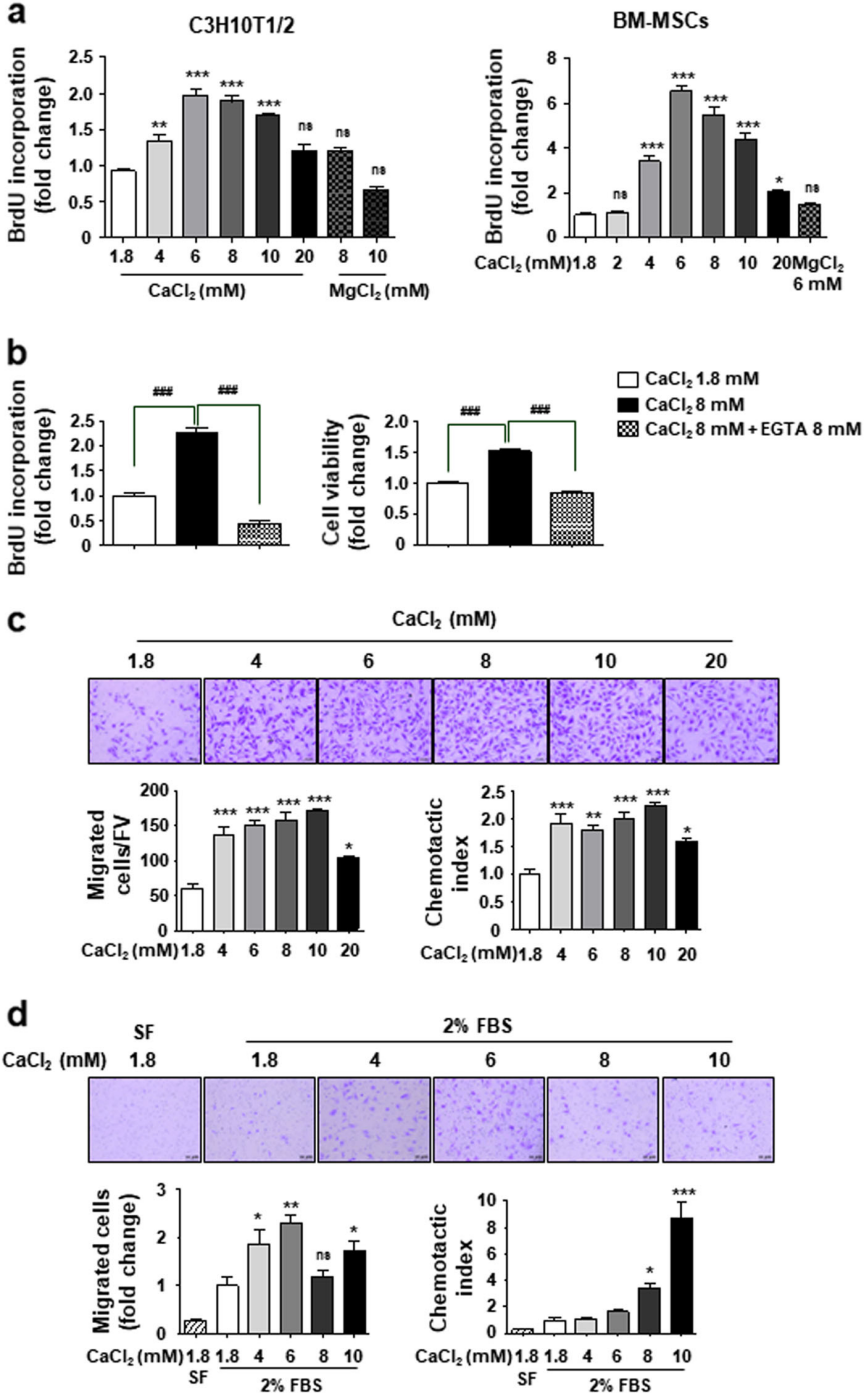
Effect of extracellular Ca^2+^ on MSCs behavior. **a**, **b** Extracellular Ca^2+^ promotes cell proliferation in a concentration-dependent manner. Cells were cultured in 2% FBS-containing medium for 48 h with the indicated amounts of either CaCl_2_ or MgCl_2_. EGTA was used as a Ca^2+^ chelator. Cell proliferation was measured by the BrdU incorporation assay and cell viability was determined by CCK-8 assay. **c**, **d** Extracellular Ca^2+^ promotes cell migration in a concentration-dependent manner. C3H10T1/2 cells (**c**) and BM-MSCs (**d**) were used in Transwell assay with the indicated amounts of CaCl_2_-containing medium in the lower chamber. Cells that migrated to the lower side of the well for 24 h were fixed and stained. The number of migrated cells per field of view (FV, x50 magnification) is indicated (bottom left in **c**). The number of migrated cells was expressed as the fold increases relative to control (bottom left in **d**). The migration capacity of cells was expressed as the chemotatic index by correcting the effect of Ca^2+^ on the proliferation of migrated cells during the 24 h treatment (chemotatic index = migrating cell number/fold change of proliferating cells, bottom right). SF (serum-free medium), negative control. *p < 0.05; **p < 0.01; ***p < 0.001 vs. first bar, ^#^*p* < 0.05; ^##^*p* < 0.01; ^###^*p* < 0.001 vs. indicated group

Ca^2+^ concentrations reach as high as 40 mM at the bone resorption site^14^. Therefore, cells were treated with culture medium containing diverse Ca^2+^ concentrations ranging from 1.8 mM up to 40 mM. However, due to cellular toxicity above 30 mM, we used 20 mM as the highest extracellular Ca^2+^ concentration in this study. Proliferation of both C3H10T1/2 cells and primary BM-MSCs were promoted at the same range of extracellular Ca^2+^ concentration and this effect reached a maximum of 6 mM concentration and thereafter gradually declined (Fig. 1a). Treatment with 6 mM Mg^2+^ or co-treatment with 8 mM Ca^2+^ and 8 mM EGTA (Ca^2+^ chelator) had no effect (Figs. 1a, b), suggesting the specific role of extracellular Ca^2+^ on MSCs proliferation.

We examined whether elevated extracellular Ca^2+^ affects the migration of MSCs in the Transwell assay. We found an increase in migrated cell numbers under elevated extracellular Ca^2+^ concentrations in both C3H10T1/2 cells and BM-MSCs (Figs. 1c, d, bottom left). Since elevated extracellular Ca^2+^ promotes cell proliferation (Fig. 1a), we further calculated the migration of cells by correcting the effect of proliferation on the number of migrated cells during the 24 h treatment; The migration capacity (chemotactic index) increased two-fold in C3H10T1/2 cells and eight-fold in BM-MSCs (Figs. 1c, d, bottom right). There was little effect of high concentration of extracellular Mg^2+^ and Ca^2+^ on attachment and spreading of MSCs (Supplementary Figure 1).

There are controversial reports on the effect of elevated extracellular Ca^2+^ on osteogenic differentiation and matrix mineralization^21,23,27^. We found that matrix mineralization was significantly enhanced by the elevated extracellular Ca^2+^. Interestingly, this effect was sustained up to 20 mM Ca^2+^ in growth medium conditions, whereas decreased after 6 mM Ca^2+^ concentration under osteogenic induction conditions (OM) (Supplementary Figure 2a). Extracellular Mg^2+^ had little effect on matrix mineralization (Supplementary Figure 2a). From these results, we suggest that elevated extracellular Ca^2+^ potentiates matrix mineralization even in the absence of osteogenic factors, and this effect was limited to the specific range of Ca^2+^ concentrations under osteogenic induction conditions.

### Extracellular Ca^2+^ concentration induces the expression of FGF2, TGFβ1, and OPN in MSCs

**Fig. 2:**
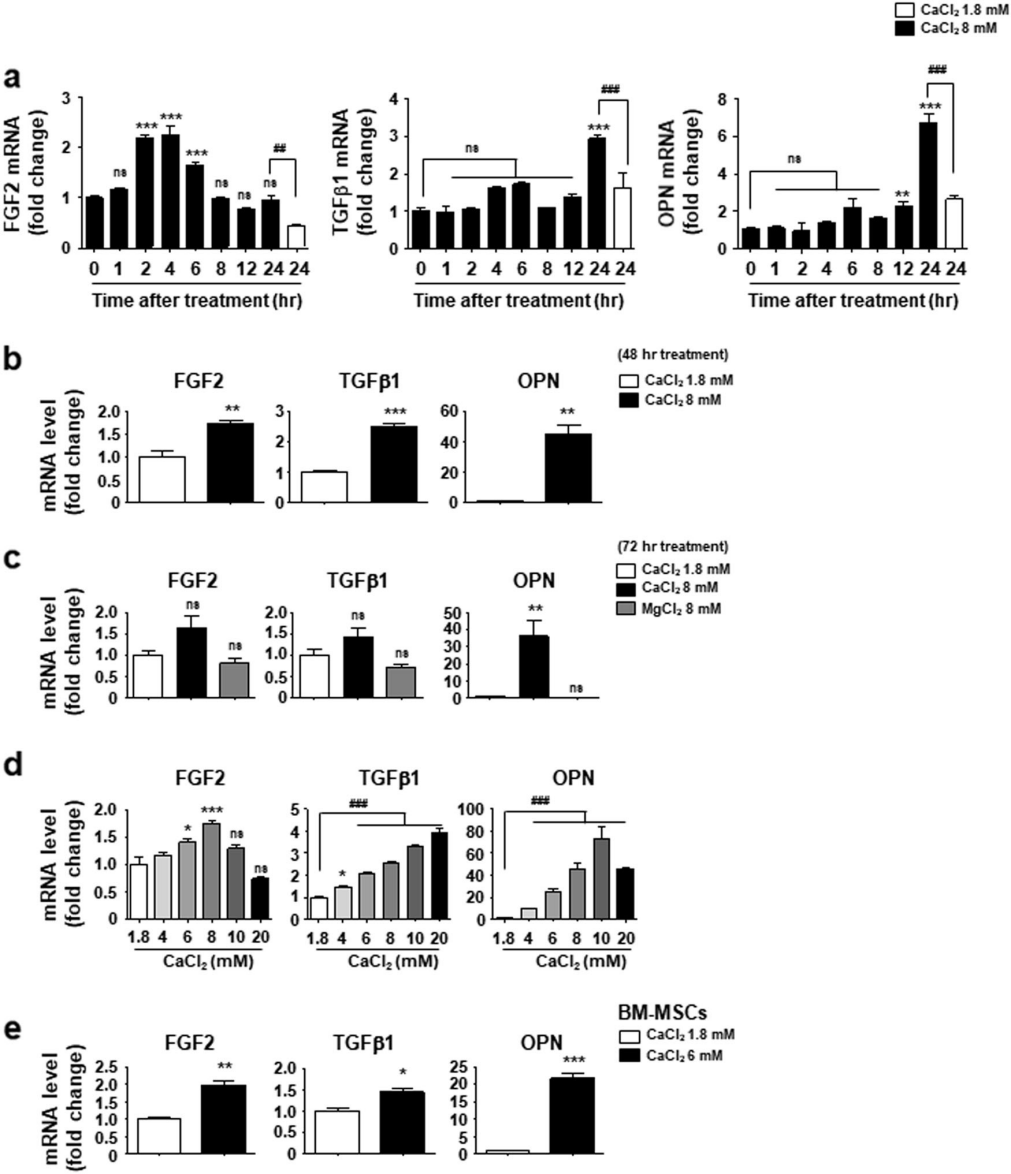
Elevated extracellular Ca^2+^ induces the expression of FGF2, TGFβ1 and OPN in a time and dose-dependent manner. **a-c** Time courses of FGF2, TGFβ1, and OPN expression after exposure to 8 mM Ca^2+^. C3H10T1/2 cells were incubated with standard medium (1.8 mM Ca^2+^) or 8 mM Ca^2+^ medium for the indicated time (**a**); 48 h (**b**); 72 h (**c**). **d** C3H10T1/2 cells were treated with a medium containing the indicated concentration of Ca^2+^ for 48 h. **e** BM-MSCs were incubated with standard medium or 6 mM Ca^2+^ medium for 48 h. **a**–**e** Cells were harvested and subjected to real-time RT-PCR to analyze expression levels of FGF2, TGFβ1, and OPN. Values for the relative expression of the indicated genes are expressed as the mean ± SEM of triplicate reactions in one representative experiment. **a**, **c**, and **d** Statistical analysis was performed by ANOVA followed by the Tukey post hoc test. **p < 0.01; ***p < 0.001 vs. first bar. ^##^*p* < 0.01; ^###^*p* < 0.001 vs. indicated group. **b** and **e** Statistical analysis was performed by Student’s *t*-test, *p < 0.05; **p < 0.01; ***p < 0.001 vs. first bar

To investigate potential factors underlying the potent role of extracellular Ca^2+^ in MSCs, we examined the expression levels of several growth factors and transcription factors that are known to play major roles in MSCs function. Cells were treated with either 6 or 8 mM Ca^2+^, the optimal concentration to alter MSC phenotype (Fig. 1a), in order to mimic the condition with elevated extracellular Ca^2+^. We found a significant increase in FGF2, TGFβ1, and OPN expression under elevated extracellular Ca^2+^ (Supplementary Figure 3). FGF2 expression increased after 2 h of treatment, and no longer responded at 72 h (Figs. 2a–c). TGFβ1 expression induced after 24 h of treatment was sustained up to 48 h (Fig. 2a, b), when the expression also showed a Ca^2+^ concentration dependency (Fig. 2d). No TGFβ1 induction occurred after 72 h post-treatment (Fig. 2c). The OPN expression induced at 12 h increased dramatically up to 72 h after treatment (Figs. 2a–c). OPN expression reached a maximum at 10 mM concentration and subsequently declined (Fig. 2d). The effect of extracellular Ca^2+^ on FGF2, TGFβ1, and OPN gene expression was also observed in BM-MSCs (Fig. 2e). Based on these results, we suggest an intrinsic regulation of the expression of these three factors in response to the changes in extracellular Ca^2+^ concentration, although their gene expression patterns vary slightly.

### MSCs secrete OPN under the elevated extracellular Ca^2+^ conditions

**Fig. 3:**
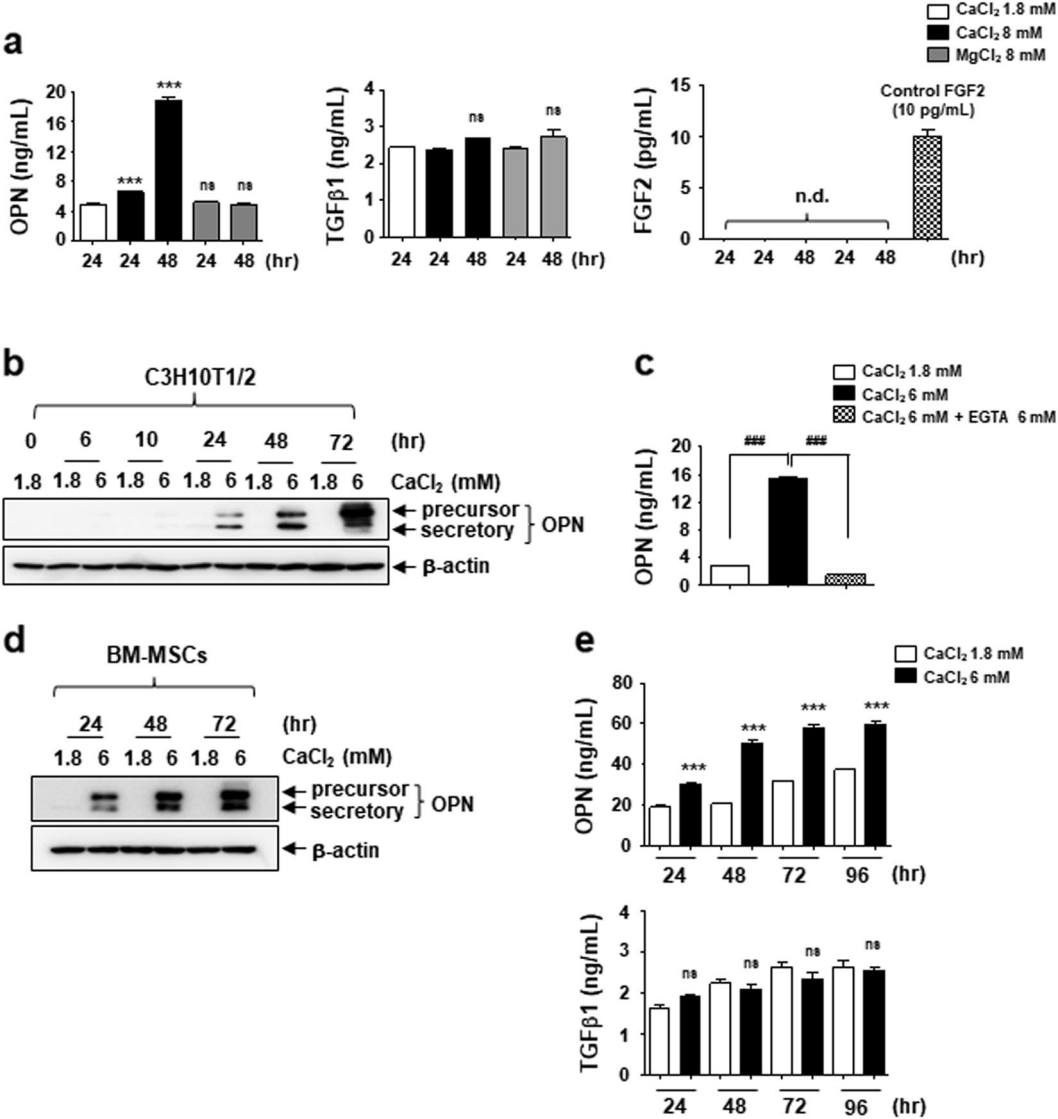
Extracellular OPN is increased by MSCs under elevated extracellular Ca^2+^ conditions. **a** Measurement of extracellular OPN, TGFβ1, and FGF2 in the indicated conditioned medium. Conditioned medium was harvested from different cultures of C3H10T1/2 cells as indicated and used to ELISA as described in the Materials and methods section. **b** and **d** Time course of effect of elevated extracellular Ca^2+^ on OPN protein levels. C3H10T1/2 cells (**b**) and BM-MSCs (**d**) were treated with 6 mM Ca^2+^ medium and harvested at the indicated time points followed by Western blotting analysis. **c** The expression and secretion of OPN is induced by elevated extracellular Ca^2+^ specifically. Extracellular OPN levels were analyzed by ELISA after the C3H10T1/2 cells were incubated for 72 h as indicated. **e** Extracellular OPN and TGFβ1 level in the conditioned medium derived from different cultures of BM-MSCs as indicated. ***p < 0.001 vs. first bar. ^###^*p* < 0.001 vs. indicated group. *ns* not significant; *nd* not detected

Because FGF2, TGFβ1, and OPN are secretory factors, we investigated the increase in their levels in the conditioned medium from cells stimulated by elevated extracellular Ca^2+^. Consistent with data from Fig. 2, a high level of OPN was detected in the conditioned medium at 48 h treatment (Fig. 3a). While active TGFβ1 was detected in all conditioned media, there was no further increase in the elevated extracellular Ca^2+^-conditioned medium (Fig. 3a). We could not evaluate the secretion of FGF2 by the stimulated MSCs, because FGF2 was rarely detected in the conditioned media (Fig. 3a). We presumed that the amount of FGF2 in the conditioned media was less than the levels of the minimum detectable dose (<1 pg/mL).

Using Western blot and ELISA assays, we confirmed that elevated extracellular Ca^2+^ increased OPN expression and secretion (Figs. 3b, c). Both precursor and secretory forms of OPN showed a continually increased expression after 24 h treatment in C3H10T1/2 cells (Fig. 3b). Expression of OPNs was also dramatically induced by elevated extracellular Ca^2+^ in BM-MSCs, but was not further increased after 48 h of treatment (Fig. 3d). In parallel, the level of OPN in the conditioned medium peaked at 48 h and maintained up to 96 h in BM-MSCs (Fig. 3e). No increase occurred in TGFβ1 levels (Fig. 3e). Although FGF2 and TGFβ1 expressions were induced by elevated extracellular Ca^2+^, this may not be sufficient to alter the extracellular levels of these proteins. From these results, it appears that OPN meets the requirement as an autocrine or paracrine factor for MSCs under the elevated extracellular Ca^2+^ conditions.

### The secreted OPN by elevated extracellular Ca^2+^ does not affect cell proliferation and matrix mineralization

**Fig. 4:**
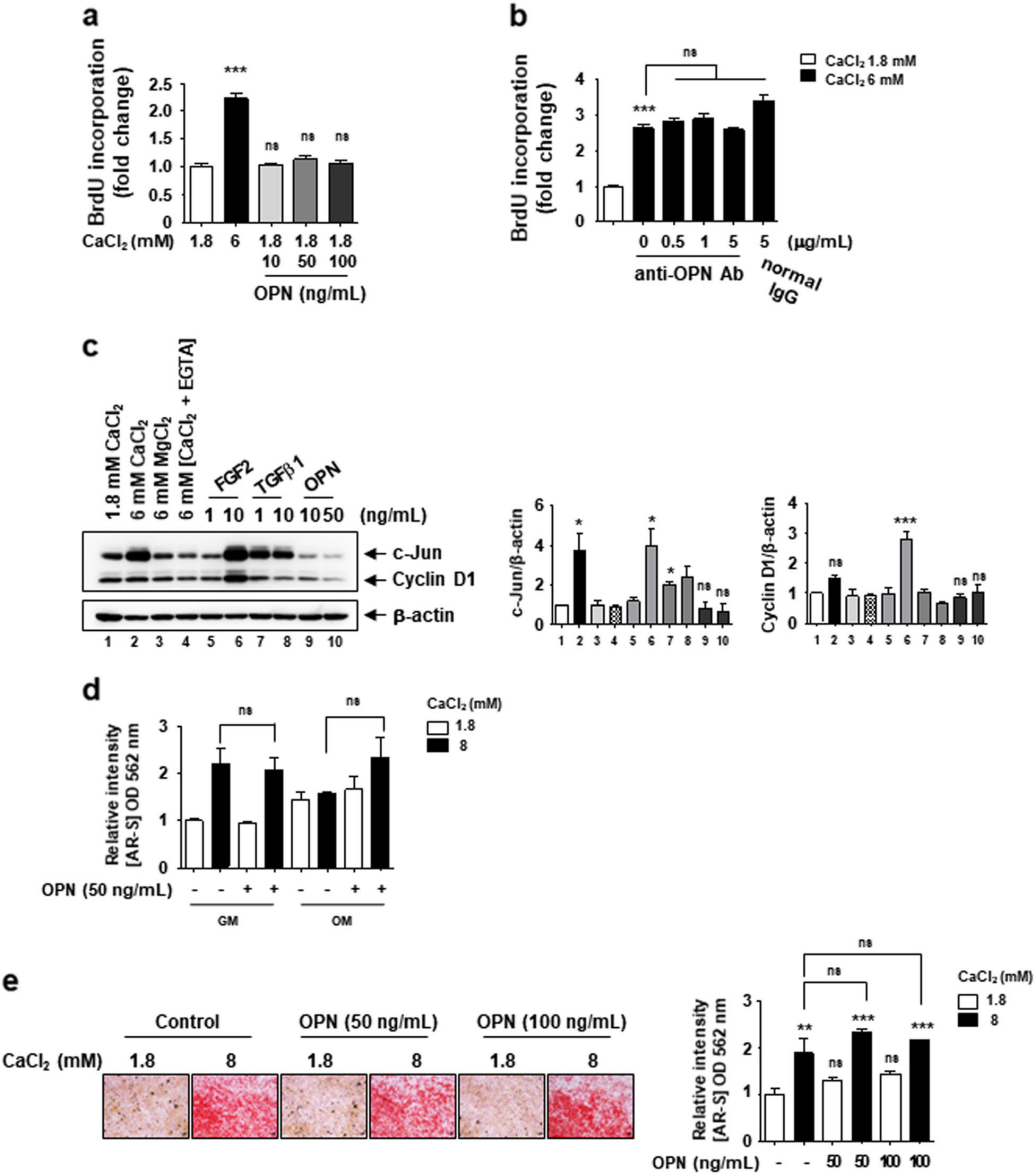
Excessive extracellular OPN induced by elevated extracellular Ca^2+^ conditions does not affect cell proliferation and matrix mineralization. **a** The effect of recombinant OPN on cell proliferation. C3H10T1/2 cells were incubated in 6 mM Ca^2+^ medium or in the standard medium with the indicated amount of recombinant OPN, and cell proliferation was measured after 48 h treatment. **b** The effect of elevated extracellular Ca^2+^-induced cell proliferation is not affected by blocking the OPN action. After 48 h of treatment with the OPN neutralizing antibody and elevated extracellular Ca^2+^ medium, a BrdU incorporation assay was performed. Normal mouse IgG antibody was used as a control for antibody treatment. **c** Elevated extracellular Ca^2+^ increase c-Jun protein level. After 48 h of treatment as indicated, the relative expression of Cyclin D1 and c-Jun was calculated after normalization to β-actin. **d** The effect of recombinant OPN supplementation on matrix mineralization. C3H10T1/2 cells were incubated in normal growth medium (GM) or osteogenic medium (OM) supplemented with the indicated concentration of CaCl_2_ and recombinant OPN. Cells were subjected to AR staining after 16 days of differentiation and staining was quantified. **e** Supplementation of excess extracellular OPN has no effect on the elevated extracellular Ca^2+^-induced matrix mineralization. C3H10T1/2 cells were incubated in GM supplemented with the indicated concentration of CaCl_2_ and recombinant OPN, and were subjected to AR staining at day 12. The bar graph shows the relative intensity of AR staining. *p < 0.05; **p < 0.01; ***p < 0.001 vs. first bar

Since OPN is a well-known cytokine that plays an important role in cell proliferation, differentiation, and migration^28–30^, we evaluated the role of the secreted OPNs on phenotype changes of MSCs under elevated extracellular Ca^2+^ conditions. We found that cell proliferation was not increased by the treatment of recombinant OPNs, while 6 mM Ca^2+^-medium potently enhanced cell proliferation (Fig. 4a). In addition, when secreted OPNs function was inhibited by adding an OPN neutralizing antibody (Fig. 4b and Supplementary Figure 4a) and also by OPN genes silencing (Supplementary Figure 4b), elevated extracellular Ca^2+^ sustains its ability to promote cell proliferation. FGF2 is a well-known growth factor promoting MSCs proliferation by inducting c-Jun and cyclin D1 expression^26,31^. We found that elevated extracellular Ca^2+^ strongly induced the levels of c-Jun, equivalent to those of the effective concentrations of FGF2 and TGFβ1 on cell proliferation (Fig. 4c and Supplementary Figure 5). However, there was no effect of OPN on both c-Jun and cyclin D1 expression (Fig. 4c), implying that OPN does not implicate in extracellular Ca^2+^-stimulated cell proliferation via c-Jun induction.

To investigate the role of the secreted OPN in matrix mineralization, cells were differentiated in osteogenic medium (OM) supplemented with recombinant OPNs. Matrix mineralization was not affected by supplementing with recombinant OPNs in both normal growth and osteogenic conditions (Fig. 4d). We also found that the supplementation of excess recombinant OPN did not enhance extracellular Ca^2+^-induced matrix mineralization (Fig. 4e). We suggest that the secreted OPN is not implicated in the promotion of cell proliferation or matrix mineralization under elevated extracellular Ca^2+^ conditions.

### The secreted OPN by elevated extracellular Ca^2+^ promotes MSCs migration

**Fig. 5:**
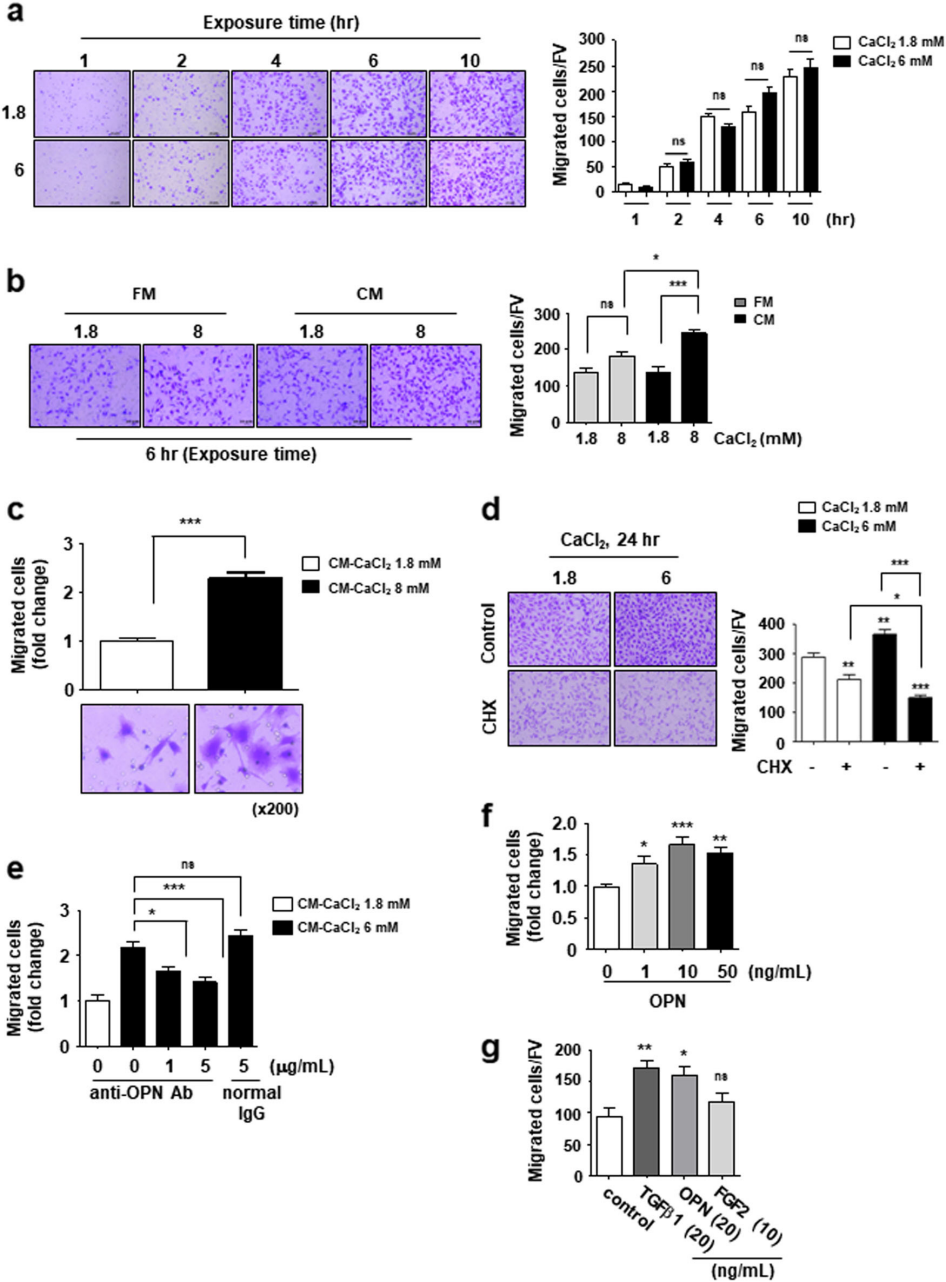
Secreted OPN from MSCs acts as a chemotactic factor for MSCs under elevated extracellular Ca^2+^ conditions. **a** Extracellular Ca^2+^ itself does not act as a cellular chemoattractant. C3H10T1/2 cells were used in Transwell assay with standard medium or 6 mM Ca^2+^ medium in the lower chamber. Cells that migrated to the lower side of the well for the indicated time were fixed and stained. **b** and **c** Conditioned medium (CM) from C3H10T1/2 cells cultured under the elevated extracellular Ca^2+^ condition induces cell migration. **b** Migration of C3H10T1/2 cells for 6 h to the lower chamber containing the indicated medium. Standard medium supplemented with the indicated concentration of CaCl_2_ (fresh medium, FM) was used as a control. **c** Migration of BM-MSCs for 6 h to the lower chamber containing in the indicated medium. **d** Migration inducible ability of CM is abolished following inhibition of new protein synthesis. C3H10T1/2 cells were seeded with or without 5 μg/mL of cycloheximide (CHX) in the upper chamber. Cells that migrated to the lower side of the well for 24 h were fixed and stained. **e-g** Extracellular OPN enhances migration of MSCs. **e** Depletion of OPN in the CM by using neutralizing antibodies abolishes the elevated extracellular Ca^2+^-induced cell migration. The indicated CM was pre-incubated with an OPN neutralizing antibody for 1 h and used as the lower chamber medium. C3H10T1/2 cells that migrated to the lower side of the well for 6 h were fixed and stained. **f** Migration of C3H10T1/2 cells for 6 h to the lower chamber containing the indicated amount of recombinant OPN in standard medium. Number of migrated cells expressed as fold increases relative to control (**e** and **f**). **g** Migration of BM-MSCs for 4 h to the lower chamber containing 0.1% BSA (control), 20 ng/mL TGFβ1, 20 ng/mL OPN, or 10 ng/mL FGF2. Number of migrated cells per field of view (FV, x50 magnification) is indicated (**a**, **b**, **d**, and **g**). Number of migrated cells expressed as the fold increases relative to control (**c**, **e**, and f). *p < 0.05; **p < 0.01; ***p < 0.001 vs. indicated group. *p* < 0.001 (**a**–**e**). *p < 0.05; **p < 0.01; ***p < 0.001 vs. first bar (**f** and **g**)

To investigate the role of the secreted OPN in the elevated extracellular Ca^2+^-induced MSCs migration, we first examined whether Ca^2+^ per se acts as a chemoattractant. Transwell assay performed for short duration in the absence of OPN secretion under elevated extracellular Ca^2+^. Cells migrated toward the lower chamber containing the indicated concentration of Ca^2+^ after 1 h exposure and the number of migrated cells were continually increased up to 10 h (Fig. 5a). However, the number of migrated cells exposed to 6 mM Ca^2+^ medium was approximately equal to that of cells exposed to the standard medium, implying that Ca^2+^ itself could not promote cell migration. When exposed to the conditioned medium (CM) obtained from 24 h-incubated cells under the elevated extracellular Ca^2+^ conditions, cells showed increased migration capacity at 6 h after treatment (Fig. 5b). The fresh medium supplemented with extracellular Ca^2+^ did not increase cell migration during this period. This effect of CM was also observed in BM-MSCs (Fig. 5c). When treated with elevated extracellular Ca^2+^ in the presence of cycloheximide, a protein synthesis inhibitor, we found that migrated cells were not increased and rather reduced when compared with control conditions (Fig. 5d). This result demonstrates again that newly synthesized proteins might involve in cell migration as well as cell proliferation. As the newly synthesized OPNs were secreted into culture medium under elevated extracellular Ca^2+^ conditions, the conditioned medium were depleted the secreted OPNs by using neutralizing anti-OPN antibodies before performing the Transwell assay. Cell migration induced by the elevated extracellular Ca^2+^-conditioned medium was significantly reduced by depletion of secreted OPNs (Fig. 5e). Moreover, the treatment of recombinant OPNs promoted cell migration of MSCs (Fig. 5f). TGFβ1 is as a potent chemokine of MSCs^12^. The degree of induction of cell migration by OPN was equivalent to that of TGFβ1 concentrations (Fig. 5g), suggesting that OPN secretion by MSCs under elevated extracellular Ca^2+^ represents a potent chemokine inducting MSCs migration. Based on these results, we concluded that elevated extracellular Ca^2+^ promotes cell migration via induction of expression and secretion of OPNs from MSCs stimulated by extracellular Ca^2+^.

### Release of calcium ions from calvarial bone slices by osteoclast-activated bone resorption affects MSCs phenotype

**Fig. 6:**
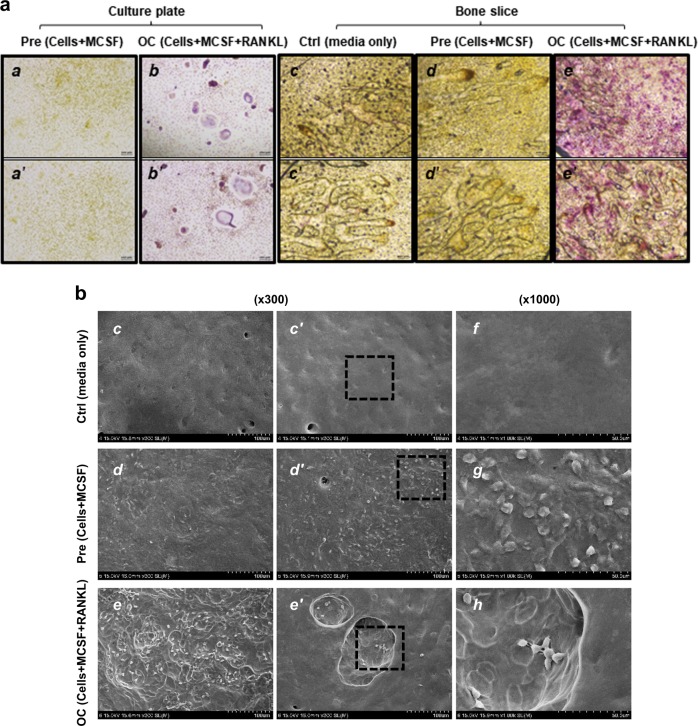

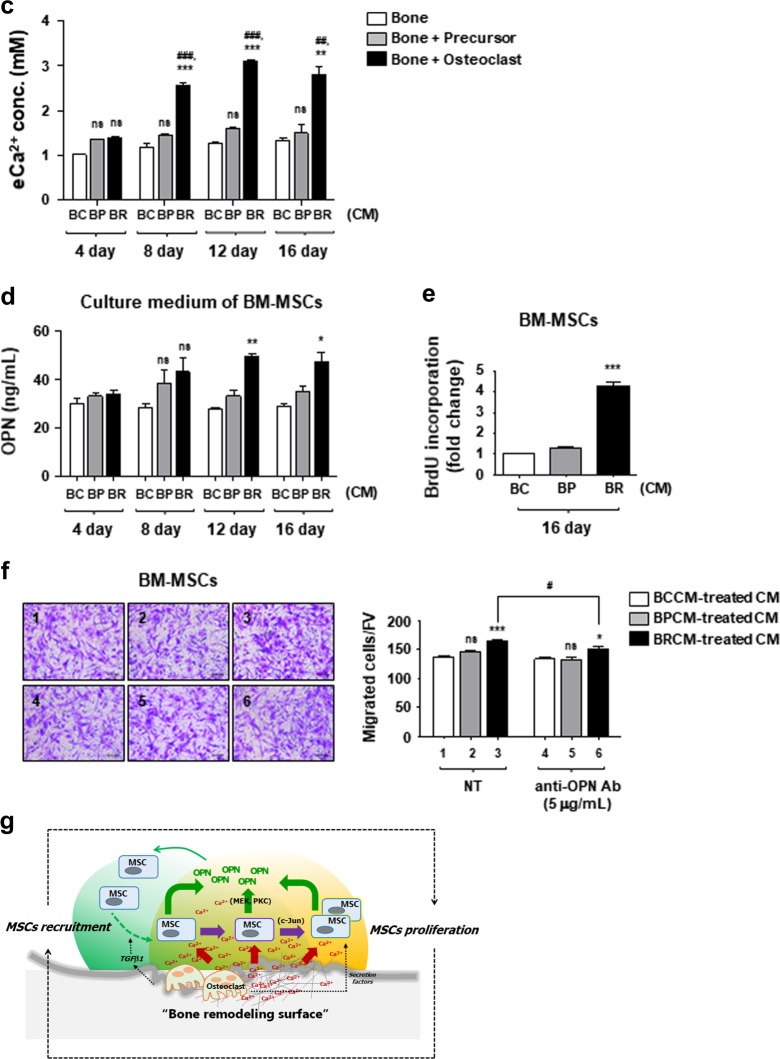
Increased extracellular Ca^2+^ in osteoclast-activated bone resorption conditioned medium affect MSCs phenotype. **a** Preparation of osteoclastic bone resorption conditioned medium. Osteoclastic precursors isolated from mouse bone marrow were cultured on mouse calvairal bone slices in the presence of MCSF and RANKL to induce osteoclast differentiation (OC), or with only MCSF as a control (Pre). To monitor morphological change of precursors and determine osteoclast differentiation, precursors were cultured under the same condition without bone slices (***a***, ***b****’*). After 16 days of incubation, cells were stained for TRAP activity. Precursors and bone slice only showed TRAP-negative (***a***, ***a****’*, ***c***, ***c****’*, ***d***, ***d****’*), and active osteoclast revealed TRAP-positive (***b***, ***b****’*, ***e***, ***e****’*). Image, x100 magnification. **b** Analysis of bone surface topology by using scanning electronic microscopy (SEM). SEM image showed a rough bone surface with several dimples by the osteoclast-activated bone resorption (***e***, ***e****’*, ***h***). Bone slice surface in the presence of media alone was clean without any cells and dents (***c***, ***c****’*, ***f***). Image, x300. The 1000x magnification images represent dotted rectangle boxes. **c** After collecting the bone resorption conditioned medium as described in Materials and methods section, the extracellular Ca^2+^ concentration was analyzed. *BCCM* bone control-conditioned medium, *BPCM* precursor with bone-conditioned medium, *BRCM* osteoclast-activated bone resorption-conditioned medium. **d** BRCM induces secretion of OPN in BM-MSCs. BM-MSCs were treated with the indicated bone resorption-conditioned media (4, 8, 12, and 16th day) for 48 h. Culture medium was collected and used for ELISA to measure secreted OPN levels. **e** BRCM increases cell proliferation of BM-MSCs. Cells were incubated in the indicated conditioned medium, and cell proliferation was measured by BrdU assay after 48 h treatment. **f** BRCM-treated culture medium (CM) enhances the migration of BM-MSCs. BRCM-treated CM from the 16th days was loaded in the lower chamber. To neutralize secreted OPN, the indicated CMs were pre-incubated with individual neutralizing antibodies for 1 h and used as the medium for the lower chamber. Migration of BM-MSCs for 6 h to the lower chamber were visualized with staining (left). Number of migrated cells per field of view (FV, x50 magnification) is expressed (right). **g** Working model illustrating the role of ionized calcium in bone remodeling surface. **p < 0.01; ***p < 0.001 vs. BCCM. ^##^*p* < 0.01; ^###^*p* < 0.001 vs. BPCM. ***p < 0.001 vs. first bar

This study was initiated by questioning whether the extracellular Ca^2+^ released from bone remodeling surface affects MSCs. Toward this end, we designed an in vitro osteoclast-mediated bone resorption assay using mouse calvaria bone slices. We confirmed that bone marrow-derived precursor cells were stably attached to calvaria bone surface (Figs. 6b: *d*, *d'* and *g*), and then successfully differentiated into osteoclasts, which was determined by TRAP staining (Figs. 6a: *e* and *e'*). In addition, SEM image analysis demonstrated that osteoclast-activated bone resorption occurred successfully on the surface of calvaria bone slices (Figs. 6b: *e*, *e'* and *h*). We detected ionized calcium in the conditioned media, more importantly, which significantly increased in the osteoclastic BRCM (Fig. 6c). Because extracellular Ca^2+^ concentration started to increase after 8 days of incubation in BRCM, we concluded that this increase resulted from osteoclast-mediated bone resorption (Fig. 6c). TGFβ1 was also released from osteoclast-activated bone slices as previously reported^12^, albeit in small amounts (Supplementary Figure 6a). FGF2 was not detected in any conditioned media (Supplementary Figure 6a). Finally, we examined whether the released Ca^2+^ from bone slices can alter MSCs phenotype. Corresponding to the data from Fig. 6c, the 12th and 16th days of BRCMs induced OPNs secretion from BM-MSCs (Fig. 6d). BRCM on day 16 was found to increase cell proliferation of BM-MSCs (Fig. 6e). These results suggest that elevated extracellular Ca^2+^ in BRCMs induces BM-MSCs to express and secrete OPN, as well as cell proliferation. Finally, BM-MSCs’ culture medium treated with the 16th days of BRCM was used for the migration assay; we named BM-MSCs’ culture medium treated with BRCM as “BRCM-treated CM”. We found that cell migration was significantly increased by the BRCM-treated CM comparing to the BCCM-treated CM (Fig. 6f). Moreover, cell migration induced by the BRCM-treated CM was significantly abolished by OPN neutralizing antibody (Fig. 6f). BRCM-treated CM from the 4th days did not promote cell migration at all (Supplementary Figure 6c). These observations suggest that expression and secretion of OPNs from MSCs stimulated by extracellular Ca^2+^ trigger MCSs migration. Altogether, we conclude that the extracellular Ca^2+^ released from bone remodeling surface can affect the MSCs phenotype during osteoclast-mediated bone resorption.

## Discussion

A continuous supply of MSCs in bone remodeling is a prerequisite for bone homeostasis. Although TGFβ1 has been known as a major factor triggering MSCs recruitment, understanding the homing and expansion of MSCs to the bone remodeling sites remains an important challenge. Here, we suggest that the ionized calcium derived from bone remodeling surface following osteoclast-mediated bone resorption is an important factor underlying the supplementation of MSCs to the bone remodeling site. We showed for the first time that Ca^2+^ is released from bone by osteoclast-mediated bone resorption, which results in the elevation of extracellular Ca^2+^ concentration leading to phenotypical changes in proximal MSCs involving cell proliferation and migration (Fig. 6g).

We found that the elevated extracellular Ca^2+^ induced expressions of not only cell growth factors, FGF2 and TGFβ1, but also the levels of cell cycle regulator, c-Jun. Therefore, we expected that these factors might mediate the elevated extracellular Ca^2+^-induced cell proliferation. However, the levels of secreted TGFβ1 and FGF2 did not increase in the conditioned medium, suggesting that their expression induced by elevated extracellular Ca^2+^ was insufficient to alter MSCs phenotype. In addition, FGF2 had little effect on MSCs proliferation at levels below 1 ng/mL (Supplementary Figure 5). Thus, we assume that TGFβ1 or FGF2 played no role in the elevated extracellular Ca^2+^-induced cell proliferation or c-Jun expression in this experiment. In the in vitro osteoclast-mediated bone resorption assay, the level of TGFβ1 and FGF2 did not significantly change in the conditioned medium (Supplementary Figure 6a). Meanwhile, MSCs exposed to the conditioned medium obtained from active osteoclasts (OCCM and BRCM) showed a dramatic increase in cell proliferation (Supplementary Figure 6b), implying that not only Ca^2+^ from bone but also other factors derived from active osteoclasts could affect MSCs proliferation. In conjunction with our finding that the elevated extracellular Ca^2+^ increases MSCs migration, we suggest the presence of a positive feedback loop involving cell proliferation and cell recruitment in the proximal microenvironment of the bone remodeling site (Fig. 6g). Extracellular Ca^2+^ and TGFβ1 derived from bone matrix and secretory factors derived from osteoclast might orchestrate MSCs recruitment and proliferation, although the molecular details of this cooperation remain to be elucidated. We believe that this is an important mechanism underlying the continuous supply of MSCs for bone remodeling. In the current study, however, we did not evaluate the comparative potency of bone-derived TGFβ1, extracellular Ca^2+^, and osteoclast-derived factors in inducing cell proliferation and cell recruitment at bone resorption sites. Since we found that MSCs proliferation is sensitive to changes in extracellular Ca^2+^ concentration (Fig. 1a) and both OPN and TGFβ1 had similar capacity to induce cell migration (Fig. 5g), we speculate that the role of elevated extracellular Ca^2+^ in homing and expansion of the MSCs might be significant in bone remodeling surface. Related to this issue, further studies are needed.

Another important finding of this study relates to the role of extracellular Ca^2+^ concentration in cell migration via OPN expression. OPN is an indicator of osteogenic differentiation due to its expression in the initial stages of matrix mineralization^32^. Meanwhile, extracellular OPN is known to interact with multiple cell surface receptors, including Integrin and CD44, resulting in cell adhesion, survival, and migration^28,33,34^. Indeed, the role of OPN in MSCs migration has been suggested in the previous study, showing the involvement of FAK, ERK, and Integrin β1 pathways^35,36^. However, there has been no report showing whether OPN expression is necessary or how its expression is regulated during MSCs migration. To our knowledge, this is the first study suggesting OPN expression is induced by extracellular Ca^2+^ to regulate cell migration. We have not investigated whether OPN induces MSCs migration via interaction with cell surface receptors under elevated extracellular Ca^2+^ conditions. Given that extracellular Ca^2+^ suppresses cell adhesion by attenuating the binding affinity of OPN to Integrin α_v_β_3_^37^, it will be worthwhile to study the OPN-mediated regulation of cell migration under elevated extracellular Ca^2+^ conditions.

OPN is abundantly secreted by MSCs^30^, but little is known about the regulation of its expression. Here, we suggest that extracellular Ca^2+^ concentration regulates OPN expression via activation of MEK and PKC pathways (Supplementary Figure 7a). Several types of cells sense extracellular Ca^2+^ via calcium sensing receptor (CaSR)^38–40^. The CaSR is a G protein-coupled receptor that is linked to several intracellular signaling cascades such as PLC-PKC pathway, calmodulin-mediated pathway, and MAPK pathway^39^. This receptor is expressed in bone sections containing osteoclasts, osteoblasts, and MSCs^38,41–43^, and conditional CaSR-knockout resulted in altered bone formation via changing in differentiation of osteoblast and osteoclast^41,44^. However, there are conflicting reports regarding the CaSR-dependent or independent response of bone cells to extracellular Ca^2+^^17,20,21^. In line with other reports^45,46^, we observed that the expression of CaSR in MSCs and pre-osteoblast (MC3T3-E1) cells is very low, and was hardly detected using RT-PCR (Supplementary Figure 7b). Moreover, CaSR silencing failed to affect the extracellular Ca^2+^-induced OPN expression (Supplementary Figure 7c). Although PKC or MEK pathway activity is required for OPN expression under elevated extracellular Ca^2+^ conditions, CaSR may not mediate its activity in MSCs. Instead, we found that polycystin 2 (PC2), a calcium channel protein, partially regulates the extracellular Ca^2+^-induced OPN expression (Supplementary Figure 7d). A recent study suggested that Connexin 43 (Cx43), known as gap junction protein, has an important role in osteogenic differentiation capacity of human MSCs in response to extracellular Ca^2+^^47^. N-type and P-type voltage-gated calcium channels (VGCCs) are also potential candidates, as they respond to extracellular Ca^2+^ and determine the osteoblast phenotype^20,48^. Therefore, we are focusing on these proteins (PC2, Cx43, and VGCCs) as candidates of relevance in altered MSC phenotype in response to extracellular Ca^2+^ concentration. Further studies are needed to fully understand how MSCs sense extracellular Ca^2+^ change at the bone remodeling site.

There are several reports showing the effect of extracellular Ca^2+^ on the osteogenic differentiation potential of precursor cells. We found that the elevated extracellular Ca^2+^ dramatically increased matrix mineralization in both growth and osteogenic conditions. However, enhanced matrix mineralization by extracellular Ca^2+^ was restricted to specific Ca^2+^ concentrations under osteogenic induction conditions. There are reports that higher ionic strength tends to increase the detachment of matrix proteins from the substrate^19,49^, suggesting that the balance between the secreted proteins by osteogenic cells and the detachment of precipitated matrix proteins by Ca^2+^ is critical for the regulation of matrix mineralization. Therefore, the differential effect of extracellular Ca^2+^ on matrix mineralization may be attributed to the role of high extracellular Ca^2+^ concentrations in segregating the precipitated matrix proteins secreted by osteoblastic cells. The question remains regarding how the elevated extracellular Ca^2+^ promote matrix mineralization in both growth and osteogenic conditions. Considering the previous study showing that OPN supplementation potentiates matrix mineralization of MSCs^30^, we surmised that matrix mineralization might be promoted by OPN induction. However, the supplementation of recombinant OPNs in either normal growth medium or osteogenic induction medium did not promote matrix mineralization (Figs. 4d, e). There are reports showing that elevated extracellular Ca^2+^ induces matrix mineralization in smooth muscle cells and in osteoblasts under the absence of osteogenic factor conditions, which were attributed to increased expression of Pit-1, Integrin β1, Connexin 43 (Cx43), Angiopoietin-1, and Angiopoientin-2^19,50^. We found that Cx43 and Pit1 expression was significantly increased by elevated extracellular Ca^2+^, whereas Integrin β1 expression was not affected (Supplementary Figure 2b). Expression of Angiopoientin-1 and −2, was not induced by elevated extracellular Ca^2+^ (data not shown). Given the finding that Pit1 and Cx43 expression was increased by elevated extracellular Ca^2+^ in both growth and osteogenic conditions (Supplementary Figure 2b), it would be worth to evaluate whether these factors can be implicated in extracellular Ca^2+^-mediated matrix mineralization in BM-MSCs. Regarding this issue, a further study is needed to address these outstanding questions.

We believe that MSCs may sense the elevated levels of extracellular Ca^2+^ for the expansion of MSCs to meet the basic requirements of bone formation. Moreover, OPN secreted from MSCs by elevated extracellular Ca^2+^ may function as a chemoattractant in recruiting MSCs to the proximal sites of bone resorption. Many studies have demonstrated the chemoattractant properties of OPN in a variety of cell types in response to injury and inflammation^51,52^. Considering the previous reports that OPN was elevated at a fracture site and in damaged bone matrix^53,54^, our finding supports the notion that OPN plays an important role in the recruitment of MSCs to the site of bone remodeling. Here, we would like to emphasize that the production of OPN can be induced by the elevated extracellular Ca^2+^-stimulated BM-MSCs, and not from the bone matrix or resident bone cells such as osteocytes. We believe that this may help to propagate the signal as to where and how many BM-MSCs are required for accurate bone remodeling by using the stimulated BM-MSCs as an intermediates. Although our findings are from in vitro experiments, we hope that this study elucidating the features and potential of BM-MSCs will provide the groundwork for future in vivo studies to facilitate the optimization of bone regeneration strategies.

## Electronic supplementary material

Supporting Information

Supplementary Figure 1

Supplementary Figure 2

Supplementary Figure 3

Supplementary Figure 4

Supplementary Figure 5

Supplementary Figure 6

Supplementary Figure 7
