# Supplementary material for: Elevated extracellular calcium ions promote proliferation and migration of mesenchymal stem cells via increasing osteopontin expression
Source: Exp Mol Med. 2018 Nov 5;50(11):1–16. doi: 10.1038/s12276-018-0170-6 (PMC6215840; doi:10.1038/s12276-018-0170-6)
Supplement: Supplementary file 1 — Supporting Information [file 12276_2018_170_MOESM1_ESM.doc]

**Supporting Information**

**Supplementary Figure 1. Extracellular Ca^2+^ has no effect on cell attachment and spreading.** C3H10T1/2 cells were seeded on the culture plate with the indicated concentration of CaCl_2_ or MgCl_2_, and time course of attachment and spreading were analyzed by observing the morphological changes in cells. Images obtained with light microscope at 100x magnification. Scale bars: 5 μm.

**Supplementary Figure 2. Increased extracellular Ca^2+^ promotes matrix mineralization. (a)** C3H10T1/2 cells were treated with either growth medium (GM) or osteogenic medium (OM) containing the indicated amounts of either CaCl_2_ or MgCl_2_. Cells were subjected to alizarin red (AR) staining after 16 days of differentiation. * *p*<0.05 vs. first bar, # *p*<0.05; ## *p*<0.01 vs. indicated group. **(b)** C3H10T1/2 cells were treated with either growth medium (GM) or osteogenic medium (OM) containing the indicated amounts of CaCl_2_. Expression levels of the indicated genes were analyzed by real-time RT-PCR after 8 days of differentiation. *** *p*<0.001 vs. indicated group.

**Supplementary Figure 3. Effect of elevated extracellular Ca^2+^ on the expression of several growth factors and transcription factors.** C3H10T1/2 cells were incubated with standard medium or 8 mM Ca^2+^ medium for 24 hours, harvested and subjected to real-time RT-PCR to analyze the expression levels of the indicated genes. *** *p*<0.001 vs. standard medium.

**Supplementary Figure 4. (a)** The effect of co-incubating of the OPN neutralizing antibody with elevated extracellular Ca^2+^ medium on the levels of OPN expression in cell lysates and the levels of secreted OPNs in the culture medium. C3H10T1/2 cells were treated with the indicated culture medium for 48 h. Cells were harvested and subjected to RT-PCR to analyze expression levels of OPN (left), and the culture media were collected and used for ELISA (right). The culture medium was also incubated with Protein A/G agarose beads to collect the antibody. OPN neutralizing antibody added to the conditioned medium was visualized by using SDS-PAGE and Ponceau S staining (bottom). ** *p*<0.01; *** *p*<0.001 vs. first bar. **(b)** Elevated extracellular Ca^2+^ promotes cell proliferation of OPN-silenced cells. C3H10T1/2 cells transfected with siRNA specific for mouse OPN (Santa Cruz Biotechnology). After 4 hours of incubation with the siRNAs–liposome complex, the cells were changed with standard media or high extracellular calcium media. After 48 hours, cell proliferation was measured using the BrdU incorporation assay. Control non-targeting siRNA (AccuTarget negative control siRNA, Bioneer) was used as a si-control. *** *p*<0.001 vs. indicated group.

**Supplementary Figure 5. Effect of extracellular Ca^2+^, recombinant FGF2, recombinant TGFβ1, and recombinant OPN on cell proliferation.** C3H10T1/2 cells were incubated with the indicated concentration of CaCl_2_, FGF2, TGFβ1, or OPN for 48 hours. Cell proliferation was measured using the BrdU incorporation assay. * *p*<0.05; ** *p*<0.01; *** *p*<0.001 vs. first bar.

**Supplementary Figure 6. Preparation of the osteoclastic bone resorption conditioned medium**. **(a)** Analysis of TGFβ1 and FGF2 levels in the osteoclastic bone resorption-conditioned medium. Conditioned media prepared as described in Figure 6 were used in the ELISA assay to measure the level of TGFβ1 and FGF2. **(b)** Conditioned medium obtained from active osteoclasts increases cell proliferation. BM-MSCs were incubated in the indicated conditioned medium, and cell proliferation was measured by BrdU assay after 48 hours treatment. CCM: control-conditioned medium, PCM: precursor-conditioned medium, OCCM: osteoclast-conditioned medium, BCCM: bone control-conditioned medium, BPCM: precursor with bone-conditioned medium, and BRCM: osteoclast-activated bone resorption-conditioned medium. ** *p*<0.01; *** *p*<0.001 vs. first bar. ## *p*<0.01 vs. indicated group. n.d., not detected. **(c)** BRCM-treated CM from the 4^th^ days was used for the migration assay.

**Supplementary Figure 7. Extracellular Ca^2+^-induced OPN expression mediated via PKC and MAPK pathway, but not by calcium sensing receptor (CaSR).** **(a)** C3H10T1/2 cells were incubated with DMSO, 5 µM of U0126, 5 µM of SB202190 (SB), 0.2 µM of Calphostin C (Cal C), or 1 µM of W7 in the indicated amount of CaCl_2_–containing medium. After 24 hours, the OPN gene expression was analyzed using real-time RT-PCR. **(b)** The expression of CaSR was analyzed by RT-PCR. The number label on C3H10T1/2 and MC3T3-E1 cells indicates the different passage of cells (#1: low passage, #2: high passage). The number label on BM-MSCs indicates a different origin. **(c)** OPN expression is still induced by the elevated extracellular Ca^2+^ in CaSR-silenced C3H10T1/2 cells. OPN gene expression was analyzed by real-time RT-PCR. **(d)** PC2 partially involves extracellular Ca^2+^-induced OPN expression. C3H10T1/2 cells transfected with siRNA specific for the indicated genes were incubated with standard medium (SM) or 6 mM CaCl_2_-containing medium. The silencing efficiency of the indicated genes was confirmed by RT-PCR (left), and the expression level of OPN was analyzed by real-time RT-PCR (right). *** *p*<0.001 vs. indicated group. # *p*<0.05; ### *p*<0.001 vs. control.
