# Supplementary figures and images for: Elevated extracellular calcium ions promote proliferation and migration of mesenchymal stem cells via increasing osteopontin expression
Source: Exp Mol Med. 2018 Nov 5;50(11):1–16. doi: 10.1038/s12276-018-0170-6 (PMC6215840; doi:10.1038/s12276-018-0170-6)

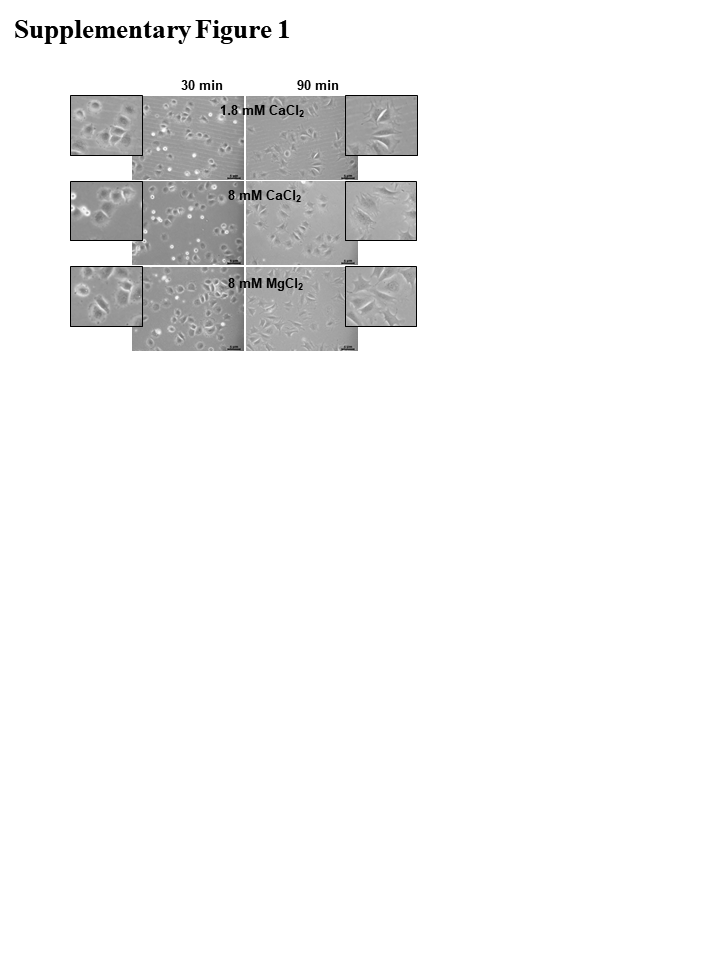

Supplement: Supplementary file 2 — Supplementary Figure 1 [file 12276_2018_170_MOESM2_ESM.tif]

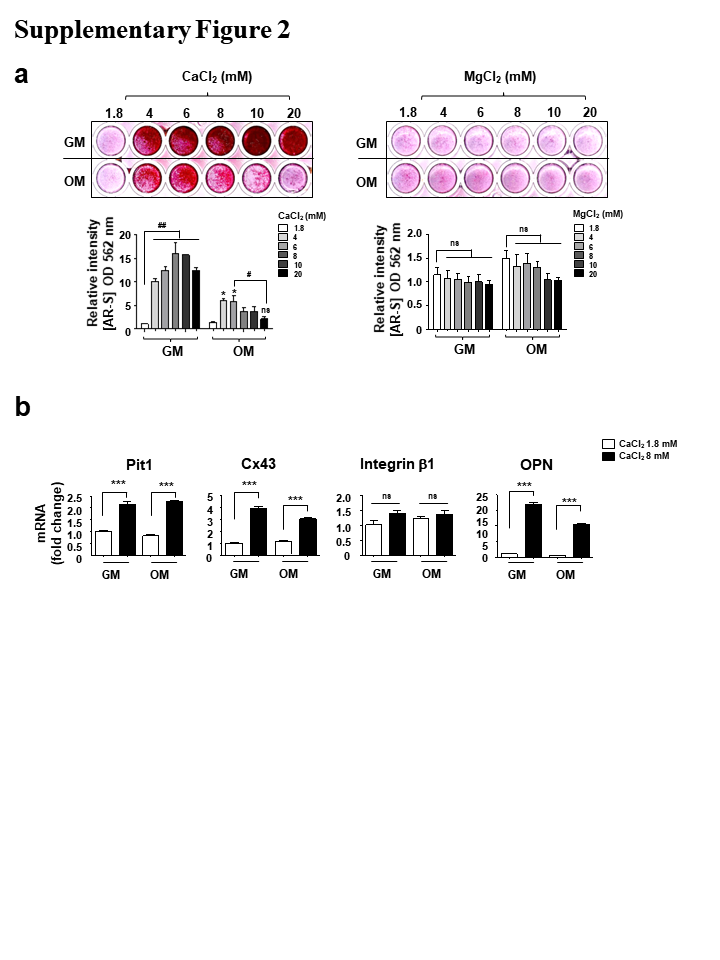

Supplement: Supplementary file 3 — Supplementary Figure 2 [file 12276_2018_170_MOESM3_ESM.tif]

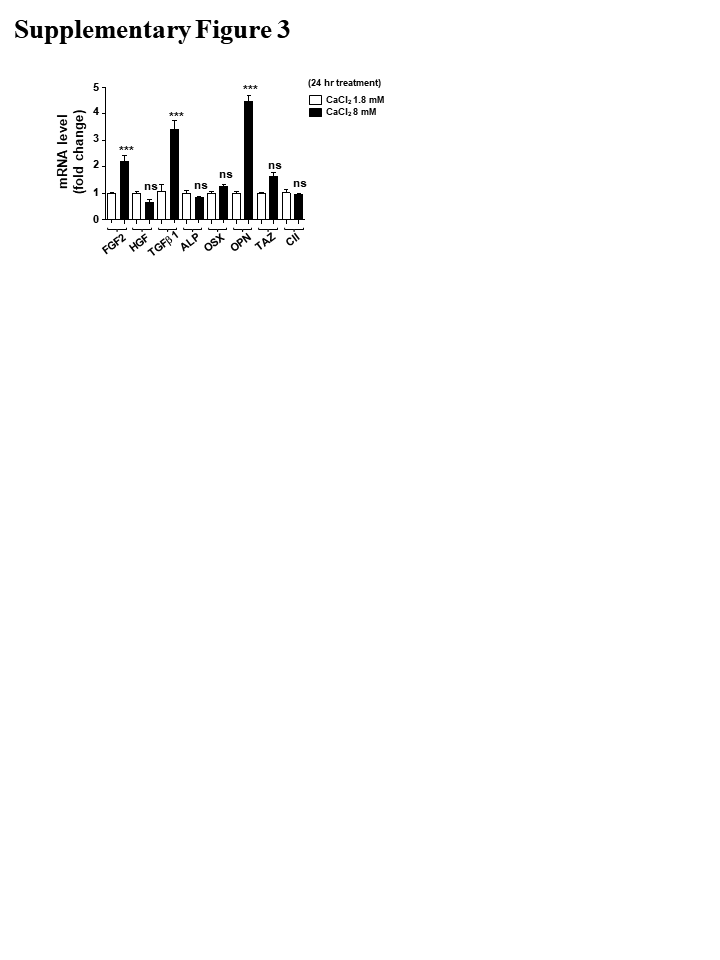

Supplement: Supplementary file 4 — Supplementary Figure 3 [file 12276_2018_170_MOESM4_ESM.tif]

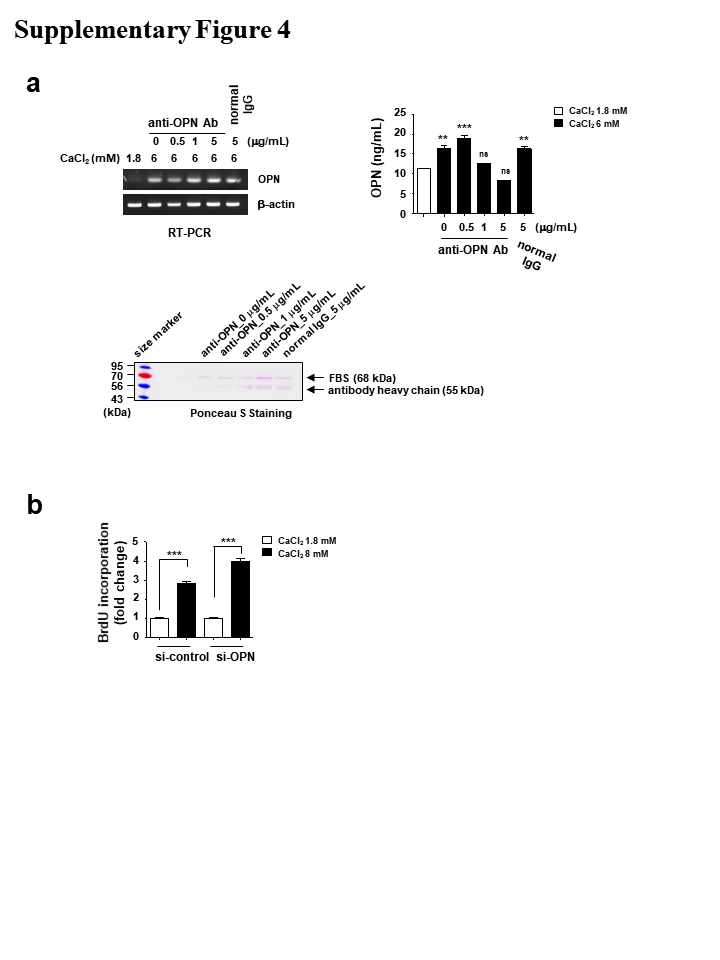

Supplement: Supplementary file 5 — Supplementary Figure 4 [file 12276_2018_170_MOESM5_ESM.tif]

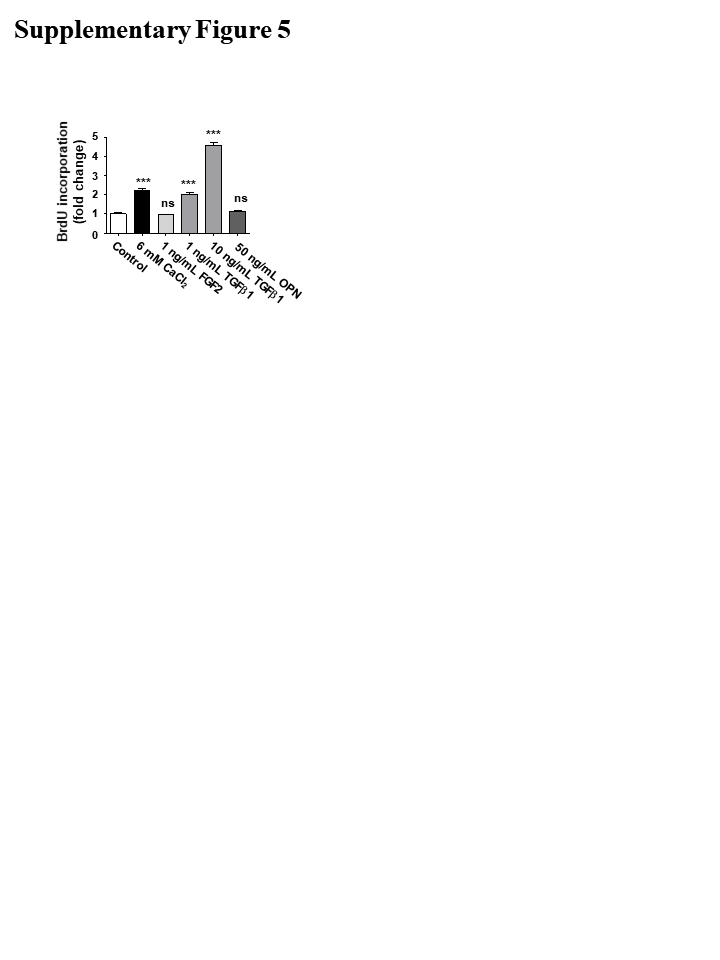

Supplement: Supplementary file 6 — Supplementary Figure 5 [file 12276_2018_170_MOESM6_ESM.tif]

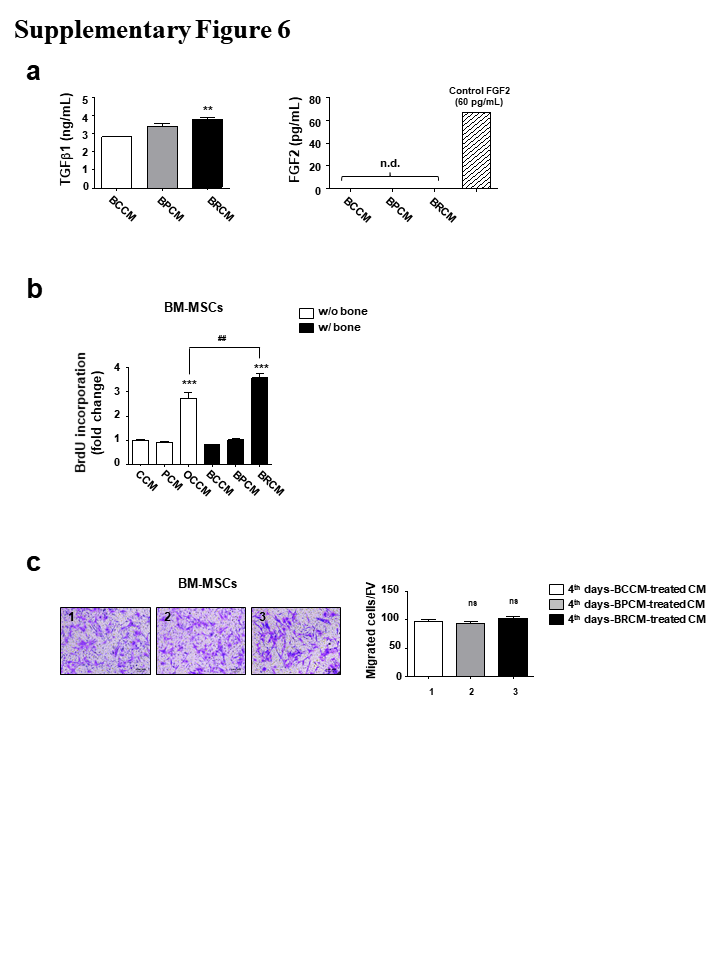

Supplement: Supplementary file 7 — Supplementary Figure 6 [file 12276_2018_170_MOESM7_ESM.tif]

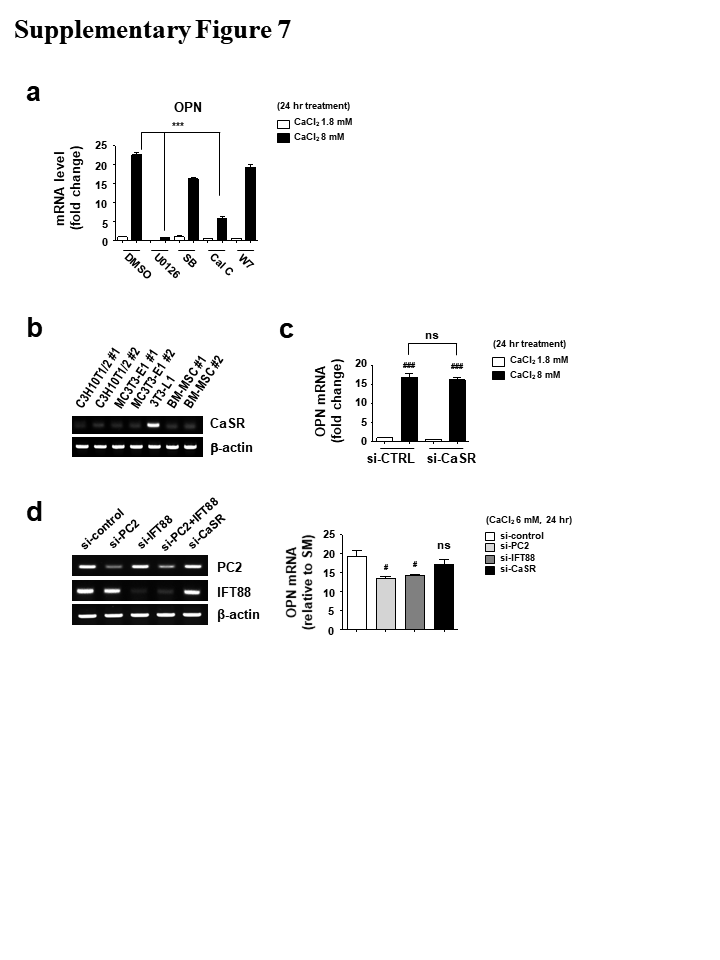

Supplement: Supplementary file 8 — Supplementary Figure 7 [file 12276_2018_170_MOESM8_ESM.tif]
